# Association between Interleukin-6 Gene Polymorphism (*rs1800795* and *rs1800796*) and Type 2 Diabetes Mellitus in a Ghanaian Population: A Case-Control Study in the Ho Municipality

**DOI:** 10.1155/2024/3610879

**Published:** 2024-04-26

**Authors:** Christian Obirikorang, Sylvester Yao Lokpo, William K. B. A. Owiredu, Linda Ahenkorah-Fondjo, James Osei-Yeboah, Kwabena Obeng Duedu, Esther Ngozi Adejumo, Samuel Ametepe, Evans Adu Asamoah, Shadrack Asiedu Coffie, Emmanuel Nattah Mawuli, Priscilla Essandoh, Precious Kwablah Kwadzokpui

**Affiliations:** ^1^Department of Molecular Medicine, School of Medicine and Dentistry, Kwame Nkrumah University of Science and Technology, Kumasi, Ghana; ^2^Department of Medical Laboratory Sciences, School of Allied Health Sciences, University of Health and Allied Sciences, Ho, Ghana; ^3^Department of Global and International Health, School of Public Health, Kwame Nkrumah University of Science and Technology, Kumasi, Ghana; ^4^Department of Biomedical Sciences, School of Basic and Biomedical Sciences, University of Health and Allied Sciences, Ho, Ghana; ^5^College of Life Sciences, Birmingham City University, City South Campus, Birmingham, UK; ^6^Department of Medical Laboratory Science, School of Public and Allied Health, Babcock University, Ilishan-Remo, Ogun State, Nigeria; ^7^Faculty of Allied Health Sciences, Koforidua Technical University, Koforidua, Greater Eastern Region, Ghana; ^8^Kumasi Centre for Collaborative Research, Kwame Nkrumah University of Science and Technology, Kumasi, Ghana; ^9^Biotechnology Laboratory, University of Ghana, Legon, Ghana; ^10^Department of Molecular Diagnostics, Claron Health International, Accra, Ghana; ^11^Laboratory Department, Ho Teaching Hospital, Ho, Ghana

## Abstract

**Background:**

There is no conclusive evidence on the association between interleukin- (IL-) 6 gene polymorphism and type 2 diabetes mellitus (type 2 DM). Thus, this study is aimed at evaluating the role of *rs1800795* and *rs1800796* polymorphisms in the pathogenesis of type 2 DM among Ghanaians in the Ho Municipality.

**Materials and Methods:**

We recruited into this hospital-based case-control study 174 patients with type 2 DM (75 DM alone and 99 with DM+HTN) and 149 healthy individuals between 2018 and 2020. Demographic, lifestyle, clinical, anthropometric, and haemodynamic variables were obtained. Fasting blood samples were collected for haematological, biochemical, and molecular analyses. Genomic DNA was extracted, amplified using Tetra-primer amplification refractory mutation system-polymerase chain reaction (T-ARMS-PCR) technique, and genotyped for IL-6 gene polymorphism. Logistic regression analyses were performed to assess the association between IL-6 gene polymorphism and type 2 DM.

**Results:**

The minor allele frequency (MAF) of the *rs1800795* and *rs1800796* polymorphisms was higher in DM alone (57.5%, 62.0%) and DM with HTN groups (58.3%, 65.3%) than controls (33.1%, 20.0%). Carriers of the rs1800795GC genotype (aOR = 2.35, 95% CI: 1.13-4.90, *p* = 0.022) and mutant C allele (aOR = 2.41, 95% CI: 1.16-5.00, *p* = 0.019) as well as those who carried the rs1800796GC (aOR = 8.67, 95% CI: 4.00-18.90, *p* < 0.001) and mutant C allele (aOR = 8.84, 95% CI: 4.06-19.26, *p* = 0.001) had increased odds of type 2 DM. For both polymorphisms, carriers of the GC genotype had comparable levels of insulin, HOMA-IR, and fasting blood glucose (FBG) with those who carried the GG genotype. IL-6 levels were higher among carriers of the rs1800796GC variant compared to carriers of the rs1800796GG variant (*p* = 0.023). The *rs1800796* polymorphism, dietary sugar intake, and exercise status, respectively, explained approximately 3% (*p* = 0.046), 3.2% (*p* = 0.038, coefficient = 1.456), and 6.2% (*p* = 0.004, coefficient = −2.754) of the variability in IL-6 levels, suggesting weak effect sizes.

**Conclusion:**

The GC genotype and mutant C allele are risk genetic variants associated with type 2 DM in the Ghanaian population. The rs1800796 GC variant, dietary sugar intake, and exercise status appear to contribute significantly to the variations in circulating IL-6 levels but with weak effect sizes.

## 1. Introduction

With recent reports suggesting an increasing prevalence of diabetes worldwide, countries in sub-Saharan Africa are expected to bear the brunt of the disease most [1] including Ghana. Thus, in 2021, the global prevalence of diabetes was estimated at 10.5%, affecting more than 536 million people aged between 20 and 79 years. This figure is expected to rise to approximately 783.2 million constituting a prevalence of 12.2% by 2045 [2]. The prevalence in Africa was projected to reach 55 million by 2045, which would be the highest percentage increase (129%) compared to other International Diabetes Federation (IDF) regions [3]. Furthermore, an estimated 416,000 deaths in the IDF African region were attributed to diabetes in 2021 [3]. In Ghana, the IDF reported that 329,200 adults aged 20-79 years, representing a prevalence of 2.0%, had diabetes in 2021 [3]. This figure may appear conservative as higher rates have been reported in older adults (3.95%) [4], and with the alarming rates of prediabetes documented among the general population [5, 6] suggest a future upsurge in the diabetes prevalence if nothing is done to reverse the trend.

Type 2 diabetes mellitus (type 2 DM) may be characterised by insulin resistance or hyperinsulinaemia [7] which predisposes to several metabolic derangements manifesting as hyperglycaemia, dyslipidaemia, abdominal obesity, and hypertension, thus increasing the risk for cardiovascular diseases [8]. In most countries around the world, rapid socioeconomic growth with changes in dietary patterns and adoption of sedentary lifestyles and less physical activity contribute to high prevalence of obesity, a major contributor to the diabetes epidemic [9], although genetic contributions cannot be discounted. It has also been suggested that chronic inflammation plays a major role in mediating vascular injury in type 2 DM. This led some researchers to believe that targeting inflammation therapeutically at the phenotypic level may help ameliorate the risk of recurrent cardiovascular events [10, 11]. Nonetheless, those assertions have been debunked by other researchers who found contradicting results [12, 13].

Over the last few decades, however, the introduction of genome-wide association studies (GWAS) made it possible to characterise the human genome and to identify its possible role in disease susceptibility. Using molecular techniques that help to identify gene mutations such as single nucleotide polymorphisms (SNPs) has led to their linkage to several diseases such that it is envisaged that genetic markers would soon be used as risk stratification tools in the clinical setting [14]. Following that, several studies have examined the association between genetic polymorphisms and susceptibility to type 2 DM including *NR1H2* gene encoding the liver X receptor beta (*LXRβ*) [15], silent mating type information regulation 2 homology (*SIRT1*) [16], *β*1-adrenegic receptor (*ADRB-1*) [17], and in particular microRNA (*miR146*) [18] and IL-6 gene polymorphisms which have been linked with putative roles in subclinical inflammation in type 2 DM. Thus, an earlier work which investigated the *rs1800795*, *rs1800796*, and *rs1800797* polymorphisms among Caucasians found an association with type 2 DM, obesity, and metabolic syndrome [19]. In a recent study, Wei et al. [20] demonstrated a significant association of rs1800796G allele with an increased risk of susceptibility to gestational diabetes, a precursor of type 2 DM in Hangzhou Fuyang, China. In Brazil, the rs1800795G allele was found to be associated with higher odds for metabolic syndrome [21]. Despite the extensive reports of GWAS investigating the role of IL-6 gene polymorphisms in type 2 DM in other jurisdictions, such studies remain scarce among Africans, and so far, only three (3) studies have been conducted, namely, among Tunisians [22], Egyptians [23], and Ethiopians [24], at the time of this study.

Moreover, findings of previous studies on the association between IL-6 genetic variants and type 2 DM have been equivocal. While some reported an association of IL-6 gene variants with insulin resistance and type 2 DM [23, 25, 26], other reported findings to the contrary [27–29], suggesting possible differences in the population characteristics that could contribute to the variations existing between studies. The paucity of similar works in the Ghanaian population and the lack of consensus on the role of IL-6 gene polymorphisms in type 2 DM represent a gap in knowledge that warranted the design of the current study. Hence, in the present study, we examined the association between rs1800795 and rs1800796 polymorphisms and type 2 DM in a Ghanaian population in the Ho Municipality of the Volta Region.

## 2. Materials and Methods

### 2.1. Study Design and Study Site

This hospital-based case-control study was conducted at the Ho Municipal Hospital in Ho in the Volta Region of Ghana. The Ho Municipal Hospital is a secondary level health facility that provides both primary and secondary level healthcare services to the people of Ho and neighboring towns including the Republic of Togo. Geographically, Ho lies between Mount Adaklu and Mount Galenukui or Togo Atakora Range. The municipality is bordered to the south with Adaklu and Agotime-Ziope districts, to the north and west with Ho West district, and to the east with the Republic of Togo. The population of the municipality based on the recent population and housing census in 2021 is 180,420, with a population density of 314.8/km^2^ and an annual population change of 0.16%.

### 2.2. Study Population and Sampling Technique

We recruited patients with type 2 DM who had been diagnosed based on the World Health Organization (WHO) criteria [30] defined as case participants and apparently healthy individuals as control participants using a convenient sampling approach. Furthermore, to be eligible as case participants, the following criteria were met: patients aged 20 years or older; diagnosed with type 2 DM with evidence of use of antidiabetic medications; without complications such as stroke, kidney disease, cardiovascular disease, cancer, thyroid disease, and infection; and not on lipid-lowering medications or hormonal therapy. Control participants were apparently healthy individuals with no history of endocrinal disorders or had infections or taking contraceptive medications recruited from the study area. Moreover, all eligible participants had fasted overnight (10-12 hours) and consented to be part of this study. However, individuals who did not meet the above-mentioned criteria were excluded from this study.

### 2.3. Sample Size Determination

The sample size for this case-control study was calculated using the G∗Power software version 3.1.9.7. Assuming an effect size, *d*, of 0.5 between the two groups (test family: *t*-tests, statistical test; means: differences between two independent means; and type of power analysis: alpha), an alpha value set at 5%, and statistical power of 95%, with allocation ratio of 1 : 1, the sample size was estimated at 210 participants. Moreover, assuming a nonresponse rate of 10% resulted in approximate sample size of 230, with a recommended minimum of 115 case participants and 115 control participants. However, 174 case participants and 149 control participants were recruited for this study.

### 2.4. Data Collection

A semistructured questionnaire was used to obtain sociodemographic data (age, gender, marital status, educational background, and type of work) and lifestyle parameters (dietary salt, sugar, and fat intake; exercise; smoking; and alcohol intake), as well as clinical variables (family history of diabetes, duration of diabetes and type of hypoglycaemic medication). Body composition indices such as BMI, body fat %, skeletal muscle mass, and visceral fat levels were measured on bioelectric impedance analysis (BIA) body composition device (Omron BF-511; Omron Healthcare Co., Ltd., Kiyoto, Japan). Waist circumference (WC) was measured in centimetres around the abdomen at the level of the umbilicus (belly button), while hip circumference (HC) was measured in centimetres as the largest circumference around the buttocks. Both WC and HC were measured using a nonextensible measuring tape. Height was measured to the nearest 0.1 cm on a stadiometer with participants standing erect, back straight, and heels together with feet slightly spread. The midupper arm circumference (MUAC) was measured on a straight left arm, midway between the tip of the shoulder and the tip of the elbow using a measuring tape. Blood pressure was measured under standard conditions with a sphygmomanometer and stethoscope by a qualified nurse. The average of two measurements made at 5-minute intervals was calculated and recorded for each participant after they had rested for 10 minutes.

#### 2.4.1. Blood Collection Procedure and Laboratory Analysis

Five millilitres (5 ml) of venous blood samples was obtained after the participants had fasted overnight (10-12 hours). Two (2) ml of the sample was placed in ethylenediaminetetraacetic acid (EDTA) tubes and stored at -80°C for DNA extraction. 1.5 ml of each remaining whole blood was dispensed into fluoride oxalate and serum separator gel tubes and centrifuged at 3000 rotations per minute. The plasma was used to estimate fasting blood glucose (FBG) levels, while the serum aliquots (in 2 ml Eppendorf tubes) were stored at -80°C and later utilised for biochemical analysis. Serum lipid profile and FBG were measured on a chemistry analyser (Selectra Pro-S Chemistry Analyser, Netherlands) according to methods provided by the reagent manufacturer (ELITech Reagents). Serum insulin and IL-6 levels were measured on an automated microplate reader (PKL Pokler, Italia) using ELISA kits (Melson Shanghai Chemical Ltd., China).

#### 2.4.2. Processing Samples for DNA Analysis

Genomic DNA was extracted based on the previously described modified salting-out technique [31]. The extraction process involved the lysis of RBCs to expose the WBCs containing genomic materials and subsequent precipitation of proteins using a highly concentrated sodium chloride solution, leaving the DNA in solution. DNA was further precipitated in absolute isopropyl alcohol (99.9%) solution, pelleted, air dried, and eluted into 50 *μ*l of nuclease-free water. The concentration and purity were estimated on a NanoDrop 1000 spectrophotometer (Thermo Scientific, Waltham, MA, USA) that yielded acceptable DNA concentrations and purities and then stored at -80°C until analysis. The Tetra primer amplification refractory mutation system (T-ARMS) polymerase chain reaction (PCR) technique was adopted in the amplification of DNA samples, and genotyping was performed using agarose gel electrophoresis.

#### 2.4.3. Primer Design for T-ARMS PCR

The primers designed for this study were based on the principle underlying the T-ARMS PCR method. Thus, two primer pairs were designed; one pair is specific to the area of amplification, and the other pair is targeted at the specific SNP in a single PCR reaction. Consequently, the reaction produces amplicons of specific band sizes that are consistent with the genotype of the sample. Two sets of primers for T-ARMS PCR were designed based on the GenBank sequence of *rs1800795* and *rs1800796* with accession numbers NC_000007.14:22727025:C:G and NC_000007.14:22726626:G:C, respectively, using a web-based software that can be accessed at http://sci.ui.ac.ir/%2Arahgozar. Several pairs of primers that were analysed took into consideration their specificities using Primer-BLAST program (http://www.ncbi.nlm.nih.gov/blast) and Oligo Analyzer 3.1 IDT (https://www.idtdna.com/calc/analyzer). Therefore, the set of primers that joined in the specific target sequence and had adequate melting temperatures (Tm) and the lowest probability of forming secondary structures was selected for this study.

#### 2.4.4. DNA Amplification and Genotyping

We employed the T-ARMS PCR technique for DNA amplification. T-ARMS is a simple and cost-effective technique that requires four primers; one pair (forward outer and reverse primers) is combined to amplify the non-allele-specific region of the gene yielding a larger control fragment containing the site of mutation. This product serves as a template for the forward inner and reverse primers to amplify specific mutation sites to produce allele-specific smaller fragments corresponding to the wild and mutant alleles (Table 1).

Prior to PCR analysis, DNA samples were further diluted to 120 *μ*l. A total of 323 samples comprising 174 cases and 149 controls were included in the PCR analysis in a thermocycler (Bio-Rad PTC-220, Dyad MJ Research). The PCR reaction “cocktail” was set up as follows: a total reaction volume of 25 *μ*l consisting of 5.5 *μ*l nuclease-free water, 12.5 *μ*l OneTaq Quick-Load 2X Master Mix with standard buffer (Biolabs, New England), 0.5 *μ*l each of the four primers, 1 *μ*l MgCl_2_, and 4.0 *μ*l DNA template. The reaction conditions include initial denaturation at 95°C for 3 minutes, final denaturation of 35 cycles at 94°C for 45 seconds, annealing at 63°C for 1 minute, initial extension at 72°C for 2 minutes, and final extension at 72°C for 7 minutes. The PCR products were resolved on 1.5% (*w*/*v*) agarose gel in 1× TAE buffer (40 mM Tris acetate, 1 mM EDTA, and pH 8.0) at 80 V for 1.5 hours for SNP genotyping with a 1 kbp plus ladder.

### 2.5. Definition of Lifestyle Parameters

Participants were deemed to consume salt, sugar, and fat when they responded “yes” to adding moderate or high amount of salt, sugar, and red meat (used as a proxy for saturated fat consumption), respectively, to their meals on regular basis. Alcohol intake and smoking status were defined as a “yes” response to having been a current or previous heavy alcohol drinker or smoker. Regular exercise was defined as a “yes” response to engaging in activities including carrying light loads, bicycling, walking to travel, for recreation, sport, exercise, or leisure on daily basis [32].

### 2.6. Statistical Analysis

Data were generated in Microsoft Excel Spreadsheet version 2013, cleaned, and exported to a statistical software (IBM Statistical Package for Social Sciences version 26.0 (SPSS Inc., Chicago, USA (http://www.spss.com)) for analysis. Normality tests were performed on all continuous variables using the Kolmogorov-Smirnov test. Continuous variables were summarized as mean ± standard deviation, while categorical variables were summarized as frequencies and proportions. Groups of continuous variables were compared using one-way analysis of variance (ANOVA) with the Tukey-Kramer post hoc test for multiple comparisons where appropriate. Groups of categorical variables were compared using chi-square test or the Mann–Whitney test where appropriate. Pearson's correlation analysis was used to describe the relationship between study parameters. The association between IL-6 polymorphisms and type 2 DM was evaluated under the genotypic and allelic models using univariate and multivariate logistic regression analysis adjusted for covariates and cofactors such as age, dietary sugar intake and educational level, type of work, anthropometric indices, and systolic and diastolic blood pressures. These variables were included in the model because their levels differed significantly among the three sample groups. However, for both polymorphisms, we could not perform the Hardy-Weinberg equilibrium analysis owing to the low frequency or the absence of the recessive CC genotype. The generalised linear model analysis was performed to assess the effect of IL-6 gene polymorphisms on circulating levels of IL-6.

### 2.7. Ethical Consideration

Ethical clearance was sought from the Committee on Human Research Publication and Ethics (CHRPE) of the Kwame Nkrumah University of Science and Technology with protocol ID CHRPE/AP/538/17. The management of Ho Municipal Hospital granted approval for this study. Informed consent was obtained from all eligible participants and participation was voluntary.

## 3. Results

As shown in Table 2, the anthropometric and blood pressure indices were compared between the three groups. Weight (kg), BMI (kg/m^2^), visceral fat levels, and blood pressure indices (systolic and diastolic) differed significantly between the three groups (*p* < 0.05). WC was significantly higher among participants with DM and HTN compared with nondiabetic controls (*p* value < 0.001) and those with DM only (*p* < 0.001). Similarly, HC, MUAC, and body fat % were significantly higher among the DM and HTN groups compared to the controls and DM groups. However, skeletal muscle (%) was significantly lower in participants with DM and HTN compared to controls and those with DM alone.

As shown in Table 3, the mean levels of TG and VLDL-C were higher among controls compared to the case groups, while FBG levels were lower among controls compared to the cases. Fasting insulin and HOMA-IR levels were significantly higher among the case groups compared to the controls. The mean total cholesterol level was higher in controls than individuals with DM alone (*p* = 0.032).

**Figure 1:**
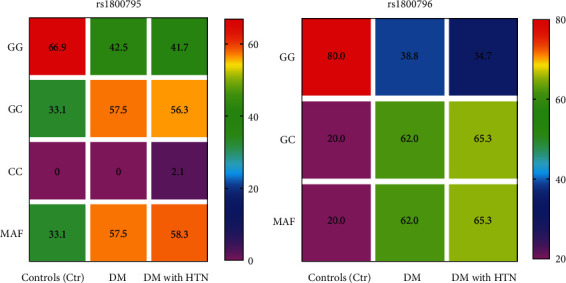
Genotype and allele frequency of IL-6 SNPs among the study participants.

Figure 1 presents the genotype and allele frequencies of IL6 SNPs among the study groups. The CC recessive genotype of *rs1800795* was absent in the controls and DM, but present among 2.1% of DM with the HTN group. The minor allele frequency (MAF) of *rs1800795* was significantly higher in the DM alone and DM with the HTN groups (57.5% and 58.3%, respectively) compared to controls (33.1%). Furthermore, the heterozygote GC genotype was disproportionately distributed between the control and diabetic groups. Unlike rs1800795, the homozygote recessive genotype of *rs1800796* was absent in the three groups, whereas the heterozygote GC genotype was present in high proportions in the diabetic groups compared to controls.

The association between IL-6 SNPs and the risk of diabetes is shown in Table 4. In the univariate model, the heterozygote GC genotypes of *rs1800795* and *rs1800796* were 2.7 and 7.1 times more likely to be present in diabetics than controls. These associations were significant after adjusting for covariates including age, gender, dietary sugar intake, educational status, and work status as well as anthropometric variables and systolic and diastolic blood pressures. In both unadjusted univariate and multivariate adjusted models, the mutant allele types of *rs1800795* and *rs1800796* (C allele) were 2 and 8 times higher in diabetics than in controls.

In Table 5, the mean FBG, insulin, and HOMA-IR were comparable between the GG and GC genotypes of the rs1800795 and rs1800796 polymorphisms (*p* > 0.05).

**Figure 2:**
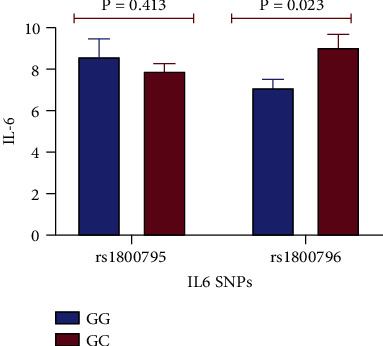
The level of IL-6 protein according to the genotypes of IL-6 polymorphism. Graph represents means with standard deviation as the error bar.

Figure 2 shows the comparison of circulating IL-6 levels between the genetic variants of the IL-6 polymorphisms. The carriers of GG genotypes had higher IL-6 levels compared to carriers of the GC genotypes of *rs1800795* polymorphism, but the difference was not statistically significant (*p* = 0.413). However, IL-6 levels of the rs1800796 GC genotypes were significantly higher compared to the rs1800796 GC genotypes (*p* = 0.023).

As shown in Table 6, the effect size of the explanatory variables in the prediction of the outcome variable (IL-6 levels) appears to be weak. The *rs1800796* polymorphism explained approximately 3% of the variability in IL-6 protein levels. Dietary sugar intake and exercise status accounted for approximately 3.2% (with a positive coefficient, 1.456) and 6.2% (with a negative coefficient, -2.674), respectively, of variability in circulating IL-6 levels.

## 4. Discussion

IL-6 single nucleotide polymorphisms have been linked with putative roles in subclinical inflammation, hypertension, and the development of type 2 DM [33]. Among the genetic biomarkers of IL-6 that have been widely studied in different populations include the *rs1800795*, *rs1800796*, and *rs1800797* variants [19–21]. Hence, the discovery of specific variants more prone to inflammation-related type 2 DM could become targets for interventions and more tailored treatment strategies. In Ghana, however, no study had previously explored the association between IL-6 gene polymorphism and type 2 DM. Thus, in this study, we sought to investigate the role of *rs1800795* and *rs1800796* polymorphisms in the pathogenesis of type 2 DM in Ho in the Volta Region. The findings would not only have implications for the clinical management of type 2 DM but provide important baseline data for future genomic studies in the study area.

Interestingly, we found that the homozygote recessive CC genotype was rare among study participants, with the carriage rate of 2.1% among participants with DM and HTN (Figure 1). The absence of the homozygote recessive CC genotype or its low frequency suggests a deviation from the Hardy-Weinberg equilibrium. The reasons for the findings are not clear, but the Hardy-Weinberg principle is violated by external forces such as extensive inbreeding, strong natural selection, low population size, mutation, and genotyping error [34, 35]. In this study, however, a strict control system was adopted to eliminate the possibility of genotyping error, as reanalysis of fifty (50) randomly selected DNA samples achieved 98% reproducibility. Moreover, rare or infrequent genetic variants have been suggested to partly explain the missing heritability of a disease [36]. Thus, we recommend large-scale population-based studies to validate the outcomes of this study. Nonetheless, previous studies have found similar results mainly among Asians, including Chinese with diabetic nephropathy (0%) [37], coal miners in Guangxi Province (0%) [38], Japanese women (0%) [39], Gujarati Indians (1.7%), and Afro-Caribbeans (0%) [40]. Furthermore, the MAF of the *rs1800795* and *rs1800796* polymorphisms was higher in participants with DM alone (57.5%, 62.0%) and those with DM and HTN (58.3%, 65.3%) compared to the nondiabetics (33.1%, 20.0%) (Figure 1). The MAF observed in the diabetic groups were higher than previously reported from a pooled data on native Africans and those of African descent (2.2%) [33], 7-39% among a Swiss cohort [41], and 23.1% among Taiwanese [37] but appears to be similar to those obtained among a US population (men vs. women; 41% vs. 40%) [27].

A major finding in this study is that IL-6 gene polymorphisms were independently associated with the risk of type 2 DM. For the *rs1800795* polymorphism, carriers of the heterozygote GC genotype (aOR = 2.35, 95% CI: 1.13-4.90, *p* = 0.022) and mutant C allele (aOR = 2.41, 95% CI: 1.16-5.00, *p* = 0.019) were twice more likely to be associated with type 2 DM, while for the *rs1800796* polymorphism, carriers of the GC genotype (aOR = 8.67, 95% CI: 4.00-18.90, *p* < 0.001) and mutant C allele (aOR = 8.84, 95% CI: 4.06-19.26, *p* = 0.001] were eight times more likely to be associated with type 2 DM compared to the homozygote dominant GG genotype and the wild-type G allele after adjustment for the covariates (Table 4). The results highlight the heterozygote GC genotype and mutant C allele as risk variants that could contribute to the pathogenesis of type 2 DM in this study cohort. In a recent past, a study conducted among Ethiopians found an association between the *rs1800795* polymorphism and type 2 DM. Thus, carriers of the GG genotype (OR = 4.61, 95% CI: 2.07-10.54) and G allele (OR = 2.81, 95% CI: 1.84-0.50) were more frequently associated with type 2 DM compared to those who carried the homozygote recessive CC genotype and mutant C allele [24] in contradiction to this study. Another study found an association of the rs1800795GC variant (OR = 1.98, 95% CI: 1.24–3.18, *p* = 0.004) and mutant C allele (OR = 1.59, 95% CI: 1.11–2.26, *p* = 0.011) with type 2 DM among a homogeneous population of Cretans in Greece, which is consistent with this study [25]. Moreover, in an earlier study, Batool et al. [42] reported a significant association between the rs1800796CC genotype and predisposition to DM onset among a Pakistani population. In contrast, the association between the *rs1800796* polymorphism and susceptibility to type 2 DM was not significant in the Bangladeshi population [28]. Most recently, a meta-analysis of 42,150 Asians demonstrated a decreased association between the *rs1800795* variants (C allele, CC and CG genotypes) and risk of type 2 DM underscoring the protective effect of the genetic variants in type 2 DM [43].

Though the genetic relationship between IL-6 and type 2 DM is complex and remains unclear till date, some reasons have been offered to explain the differences that exist between studies. These include gene-gene interactions, gene-environment interactions, differences in ethnicity or racial origin, and the general genetic makeup of the study populations [25]. Moreover, Pandaya et al. [28] opined that the association of the rs1800796GGG haplotype with *rs1800975* and *rs1800797* polymorphisms appeared to be stronger in predisposing to type 2 DM rather than the *rs1800796* polymorphism alone, although there was no evidence of linkage disequilibrium. Hamid et al. [19] appeared to hold a similar view when they posited that complex interactions between various haplotypes and the existence of ethnic specific polymorphisms in the IL6 promoter may influence IL6 gene transcription and hence its contribution to type 2 DM.

We also found that the IL-6 gene polymorphisms did not appear to play a role in the development of insulin resistance. Although carriers of the GC genotype generally had higher mean levels of glycaemic indices, including insulin resistance compared to those who carried the GG genotypes, the differences were not statistically significant (Table 5). This may be due to the absence of direct pathways linking the genetic variants with insulin resistance or that the relationship may have been masked by unrecognized confounders not measured in this study. The findings are, however, in tandem with Todendi et al. [44] who did not find an association between the *rs1800795* polymorphism and fasting glucose levels in children and adolescents and Barati et al. [45] who found no correlation between the *rs1800796* polymorphism and metabolic syndrome defined by the IDF criteria among adults with obesity. On the contrary, the findings appear to conflict with Boeta-Lopez et al. [46] who previously described a significant relationship between the rs1800796C allele and insulin resistance among young Mexican-Americans and Helaly et al. [23] who showed a significant increase in the frequency of the rs1800795 CC genotype among diabetic patients with poor glycaemic control and high insulin resistance.

The effect of genetic markers on the level of protein expression involved in the inflammatory process was apparent in this study. Carriers of the GC variant of *rs1800796* polymorphism significantly presented higher levels of IL-6 compared to those who carried the GG variant (*p* = 0.023) (Figure 2). The promoter activity of the IL-6 gene which leads to elevated expression of IL-6 levels has previously been described. According to Wei et al. [20], the G allele at the -572 locus exhibits a high level of promoter activity due to the close affinity for its cognate transcription factors that promotes increased transcriptional activity leading to high levels of IL-6. In addition, the *rs1800796* polymorphism, dietary sugar intake, and exercise status, respectively, explained approximately 3% (*p* = 0.046), 3.2% (*p* = 0.038, coefficient = 1.456), and 6.2% (*p* = 0.004, coefficient = −2.754) of the variability in IL-6 levels, suggesting weak effect sizes between the covariates and IL-6 levels (Table 6). Notwithstanding, the findings also emphasise the pro- and anti-inflammatory potentials of modifiable lifestyle parameters among the study participants. Indeed, previous studies have supported the role of excessive dietary sugar intake in the development of chronic low-grade inflammation. Using a mouse model, Zhang et al. [47] demonstrated that high dietary glucose intake activates the transforming growth factor-beta (TGF-*β*) through the reactive oxygen species (ROS) pathway that promotes Th17 cell differentiation with the participation of IL-6. In a recent review, Bodur and Unal [48] also showed that high dietary intake of fructose and saturated fatty acids could lead to the development of low-grade chronic inflammation through proinflammatory mediators. However, evidence of the anti-inflammatory effect of regular exercise has also been documented in the literature [49, 50]. Regular low-to-moderate intensity exercise has been shown to reduce IL-6 levels through mechanisms that include reduction in adipose tissue mass, development of an anti-inflammatory environment, and changes in the expression of the toll-like receptor (TLR) in innate immune cells [49].

## 5. Conclusion

The GC genotype and mutant C allele are risk genetic variants associated with type 2 DM in the Ghanaian population. The *rs1800796 GC* variant, dietary sugar intake, and exercise status appear to contribute significantly to the variations in circulating IL-6 levels but with weak effect sizes.

## Figures and Tables

**Table 1:** Primers for rs1800795 and rs1800796 genotyping.

Primer	Primer sequence (5′-3′)	Product size (bp)	Melting temperature (°C)
	*rs1800795*		
FIP	TTTTCCCCCTAGTTGTGTCTTCCG	144 (G allele)	75.4°C
RIP	GTGCAATGTGACGTCCTTTAGCTTG	181 (C allele)	74.5°C
FOP	TCGTGCATGACTTCAGCTTTACTCATT	277 (outer)	
ROP	GAGACTCATGGGAAAATCCCACTTT		
	*rs1800796*		
FIP	GGCCAGGCAGTTCTACAACAGGCG	125 (G allele)	76.8°C
RIP	TGTTCTGGCTCTCCCTGTGTGG	157 (C allele)	78.3°C
FOP	CTCTAAGTGGGCTGAAGCAGGTGATGA	237 (outer)	
ROP	AGTTTCCTCTGACTCCATCGCAGGCC		

FIP: forward inner primer; RIP: reverse inner primer; FOP: forward outer primer; ROP: reverse outer primer.

**Table 2:** Multiple comparisons of anthropometric and haemodynamic parameters between the three groups.

Variables	Controls (*n* = 149)	DM alone (*n* = 75)	DM with HTN (*n* = 99)	*p* value (a)	*p* value (b)	*p* value (c)
*Anthropometric indices*						
Weight (kg)	74.63 ± 12.00	68.60 ± 14.19	79.68 ± 14.40	**0.004**	**0.010**	**<0.001**
WC (cm)	88.50 ± 10.38	87.21 ± 13.77	95.64 ± 14.50	0.751	**<0.001**	**<0.001**
HC (cm)	99.67 ± 11.59	96.93 ± 13.88	105.19 ± 16.42	0.341	**0.006**	**<0.001**
MUAC (cm)	31.99 ± 4.15	31.19 ± 4.32	33.57 ± 4.86	0.421	**0.018**	**0.002**
BMI (kg/m^2^)	27.94 ± 4.27	25.78 ± 4.91	30.08 ± 5.46	**0.005**	**0.002**	**<0.001**
Body fat %	34.73 ± 10.93	31.61 ± 12.39	38.74 ± 10.56	0.126	**0.017**	**<0.001**
Skeletal muscle mass (%)	29.22 ± 6.23	29.77 ± 7.41	27.08 ± 5.43	0.816	**0.026**	**0.017**
Visceral fat	9.32 ± 3.02	7.92 ± 3.59	10.71 ± 2.88	**0.005**	**0.003**	**<0.001**
*Blood pressure indices*						
Systolic BP (mmHg)	127.84 ± 16.00	114.20 ± 17.20	134.13 ± 19.46	**<0.001**	**0.018**	**<0.001**
Diastolic BP (mmHg)	81.27 ± 10.03	75.20 ± 11.30	84.78 ± 11.59	**<0.001**	**0.036**	**<0.001**

Data are presented as mean ± standard deviation. *p* value (a): post hoc analysis between controls and patients with DM alone. *p* value (b): post hoc analysis between controls and DM with HTN. *p* value (c): post hoc analysis between DM alone and DM with the HTN groups. *p* < 0.05 is significant. Significant *p* values are marked in bold. Analysis was conducted using one-way analysis of variance with the Tukey-Kramer post hoc multiple comparisons. DM: diabetes mellitus; HTN: hypertension; WC: waist circumference; HC: hip circumference; MUAC: midupper arm circumference; BMI: body mass index; BP: blood pressure.

**Table 3:** Comparison of glycaemic and lipid parameters between controls, DM alone, and DM with the HTN groups.

Variables	Controls	DM alone	DM with HTN	*p* value (a)	*p* value (b)	*p* value (c)
*Lipid profile*						
TC (mmol/l)	5.51 ± 1.10	5.11 ± 1.16	5.19 ± 1.044	**0.032**	0.102	1.00
TG (mmol/l)	2.03 ± 0.81	1.41 ± 0.46	1.74 ± 0.66	**<0.001**	**0.006**	**0.007**
HDL-C (mmol/l)	1.57 ± 0.38	1.45 ± 0.39	1.38 ± 0.34	0.095	**<0.001**	0.511
LDL-C (mmol/l)	3.05 ± 1.03	3.01 ± 1.00	3.00 ± 0.99	1.00	1.00	1.00
VLDL-C (mmol/l)	0.92 ± 0.36	0.66 ± 0.26	0.80 ± 0.32	**<0.001**	**0.017**	**0.017**
*Glycaemic indices*						
FBG (mmol/l)	4.99 ± 0.60	13.54 ± 5.66	11.45 ± 4.27	**<0.001**	**<0.001**	**0.001**
Insulin (mU/l)	2.18 ± 1.70	7.49 ± 3.32	7.93 ± 3.31	**<0.001**	**<0.001**	0.908
HOMA-IR	0.47 ± 0.27	4.34 ± 2.16	3.79 ± 2.16	**<0.001**	**<0.001**	0.171

Data are presented as mean ± standard deviation. *p* value (a): post hoc analysis between controls and patients with DM alone. *p* value (b): post hoc analysis between controls and DM with HTN. *p* value (c): post hoc analysis between DM alone and DM with the HTN groups. *p* < 0.05 is significant. Significant *p* values are marked in bold. Analysis was conducted using one-way analysis of variance with the Tukey-Kramer post hoc multiple comparison test. TC: total cholesterol; TG: triglyceride; HDL-C: high-density lipoprotein cholesterol; LDL-C: low-density lipoprotein cholesterol; VLDL-C: very low-density lipoprotein cholesterol; FBG: fasting blood glucose; HOMA-IR: homeostatic model assessment for insulin resistance; DM: diabetes mellitus; HTN: hypertension.

**Table 4:** Association of *rs1800795* and *rs1800796* polymorphism with the risk of type 2 DM.

Variables	Controls	All cases	Crude model		Adjusted model	
			cOR	*p* value	aOR	*p* value
*rs1800795*						
GC vs. GG	40 (33.1)	96 (56.8)	2.74 (1.68-4.46)	**<0.001**	2.35 (1.13-4.90)	**0.022**
Allele (C vs. G)	40 (33.1)	96 (56.8)	2.80 (1.72-4.55)	**<0.001**	2.41 (1.16-5.00)	**0.019**
*rs1800796*						
GC vs. GG	29 (20.0)	108 (63.9)	7.08 (4.24-11.84)	**<0.001**	8.67 (4.00-18.90)	**<0.001**
Allele (C vs. G)	29 (20.0)	108 (63.9)	7.08 (4.24-11.84)	**<0.001**	8.84 (4.06-19.26)	**<0.001**

Data are presented as frequency with corresponding proportion in parenthesis or odd ratio with 95% confidence interval. cOR: crude odd ratio; aOR: adjusted odd ratio. Adjusted model: multivariate logistic model involving age, dietary sugar intake and educational status, work status, anthropometric indices, and systolic and diastolic blood pressures treated as covariates. *p* < 0.05 is significant. Significant *p* values are marked in bold.

**Table 5:** The levels of glycaemic indices according to IL-6 gene polymorphism.

Markers	SNP	*N*	Mean	Std. deviation	*p* value
*rs1800795 (-174G>C)*					
FBG (mmol/l)	GG	68	12.54	5.04	0.653
	GC	94	12.17	5.09	
Insulin (*μ*IU/ml)	GG	66	7.40	3.34	0.243
	GC	86	8.04	3.33	
HOMA-IR	GG	64	3.98	2.28	0.804
	GC	80	4.08	2.47	
*rs1800796 (-572G>C)*					
FBG (mmol/l)	GG	60	12.54	5.32	0.606
	GC	104	12.12	4.70	
Insulin (*μ*IU/ml)	GG	56	7.36	3.50	0.18
	GC	98	8.10	3.11	
HOMA-IR	GG	51	3.67	2.51	0.169
	GC	95	4.25	2.23	

FBG: fasting blood glucose; HOMA-IR: homeostatic model assessment for insulin resistance; SNP: single nucleotide polymorphism.

**Table 6:** Generalised linear model analysis of the effect of IL-6 polymorphism on serum levels of IL-6 protein.

Parameter	Coefficient	*T*	95% confidence interval		Partial eta squared	*p* value
			Lower bound	Upper bound		
Intercept	12.928	2.878	4.042	21.814	0.059	**0.005**
Age (years)	0.044	-0.854	-0.147	0.059	0.005	0.395
Dietary sugar intake	1.456	2.1	0.085	2.828	0.032	**0.038**
Educational status	0.474	0.825	-0.662	1.61	0.005	0.411
Work status	1.459	1.315	-0.736	3.655	0.013	0.191
Exercise status	-2.674	-2.957	-4.462	-0.886	0.062	**0.004**
TC	-0.269	-0.643	-1.096	0.558	0.003	0.521
TG	-4.347	-0.771	-15.501	6.808	0.004	0.442
HDL-C	-0.423	-0.361	-2.741	1.895	0.001	0.719
VLDL-C	7.306	0.592	-17.106	31.718	0.003	0.555
*rs1800796*	-1.768	-2.019	-3.501	-0.036	0.030	**0.046**

TC: total cholesterol; TG: triglyceride; HDL-C: high-density lipoprotein cholesterol; VLDL-C: very low-density lipoprotein cholesterol. *p* < 0.05 is significant. Significant *p* values are marked in bold.

## Data Availability

The data used for this study are available from the corresponding author upon request.

## Supplementary Materials

Supplementary Materials sTable 1: basic demographic characteristics of study participants. sTable 2: comparison of lifestyle characteristics between cases and controls. sF1 A&B: comparison of treatment duration (A) and treatment option (B) between type 2 DM and 2 DM with HTN groups.
