# Supplementary material for: Association between Interleukin-6 Gene Polymorphism (rs1800795 and rs1800796) and Type 2 Diabetes Mellitus in a Ghanaian Population: A Case-Control Study in the Ho Municipality
Source: Biomed Res Int. 2024 Apr 26;2024:3610879. doi: 10.1155/2024/3610879 (PMC11068456; doi:10.1155/2024/3610879)
Supplement: Supplementary Materials — sTable 1: basic demographic characteristics of study participants. sTable 2: comparison of lifestyle characteristics between cases and controls. sF1 A&B: comparison of treatment duration (A) and treatment option (B) between type 2 DM and 2 DM with HTN groups. [file 3610879.f1.docx]

**sTable 1: Demographic and basic characteristics of the study participants**

sTable shows the demographic characteristics of the study participants. A total of 174 participants with type 2 DM and 149 non-diabetic controls were recruited for this study. Out of the 174 diabetic participants, 75 had DM alone while 99 had both DM and HTN. The mean age was significantly higher among DM and HTN (52.2±6.4 years) and DM alone groups (46.7±8.9 years) compared to the control group (44.2±8.4 years). Unemployment rate [10 (6.7%) vs 7(7.1%)] and informal employment status [80 (53.7%) vs 73 (73.7)] were lower among controls compared to DM and HTN group. Attainment of tertiary level education was proportionally higher among the control group [51 (34.2%)] compared to the DM alone [14 (18.7%)] and DM and HTN [18 (18.2%)] groups.

**sTable 1: Basic demographic characteristics of study participants**

| **Variables** | **Controls**  **(n=149)** | **DM alone (n=75)** | **DM with HTN (n=99)** | **p-value** |
| --- | --- | --- | --- | --- |
| ***Age (years) **** | 44.2±8.4*^a,b^* | 46.7±8.9 | 52.2±6.4 | **<0.001** |
| ***Gender*** |  |  |  | 0.253 |
| Male | 60 (40.3) | 34 (45.3) | 33 (33.3) |  |
| Female | 99 (59.7) | 41 (54.7) | 66 (66.7) |  |
| ***Marital status*** |  |  |  | 0.454 |
| Single | 58 (38.9) | 24 (32.0) | 34 (34.3) |  |
| Married | 91 (61.1) | 51 (68.0) | 65 (65.7) |  |
| ***Employment status*** |  |  |  | **0.005** |
| None | 10 (6.7) *^a,b^* | 3 (4.0) | 7 (7.1) |  |
| Informal | 80 (53.7) *^a,b^* | 55 (73.3) | 73 (73.7) |  |
| Formal | 59 (39.6) | 17 (22.7) | 19 (19.2) |  |
| ***Educational background*** |  |  |  | **0.046** |
| None | 7(4.7) | 8 (10.7) | 8 (8.1) |  |
| Basic | 60 (40.3) | 38 (50.7) | 56 (56.6) |  |
| Secondary | 31 (20.8) | 15 (20.0) | 17 (17.2) |  |
| Tertiary | 51 (34.2) *^a,b^* | 14 (18.7) | 18 (18.2) |  |
| ***Family history of DM*** |  |  |  | 0.626 |
| No | 116 (77.9) | 54 (72.0) | 77 (77.8) |  |
| Yes | 33 (22.1) | 21 (28.0) | 22 (22.2) |  |

*Data presented as frequency and percentage, and comparison was done using the Chi-square test, otherwise stated. Variables with * sign are presented as mean and standard deviation and compared using ANOVA. Significant p-values are flagged in bold. ‘a’ means controls differ from DM alone significantly; ‘b’ means controls differ from DM with HTN significantly; ‘c’ means that DM alone differ significantly from DM with HTN ‘p’ means all three groups differ significantly.*

In sTable 2 shown below, the lifestyle characteristics of the three groups were compared. Self-report of moderate dietary sugar intake was predominantly higher among the control group [128 (84.6%)] compared to DM alone [30 (40.0%)] and DM with HTN [47 (47.5%)] groups whereas physical inactivity was predominantly reported among DM alone participants [23 (30.7%)] compared to the control participants [70 (47.0%)].

**sTable 2: Comparison of lifestyle characteristics between cases and controls**

| **Variables** | **Controls**  **(n=149)** | **DM alone (n=75)** | **DM with HTN (n=99)** | **p-value** |
| --- | --- | --- | --- | --- |
| ***Dietary salt intake*** |  |  |  | 0.612 |
| None | 1 (0.7) | 2 (2.7) | 2 (2.0) |  |
| Moderate | 133 (89.3) | 64 (85.3) | 91 (91.9) |  |
| High | 15 (10.1) | 9 (12.0) | 6 (6.1) |  |
| ***Dietary fat intake*** |  |  |  | 0.093 |
| None | 12 (8.1) | 12 (16.0) | 18 (12.8) |  |
| Moderate | 113 (75.8) | 54 (72.0) | 74 (74.7) |  |
| High | 24 (16.1) | 9 (12.0) | 7 (7.1) |  |
| ***Dietary sugar intake*** |  |  |  | **<0.001** |
| None | 8 (5.4) *^a,b^* | 37 (49.3) | 47 (47.5) |  |
| Moderate | 128 (84.6) *^a,b^* | 30 (40.0) | 47 (47.5) |  |
| High | 15 (10.1) | 8 (10.7) | 5 (5.1) |  |
| ***Exercise status*** |  |  |  | **0.008** |
| No | 70 (47.0) | 23 (30.7) *^a^* | 31 (31.3) |  |
| Yes | 79 (53.0) | 52 (69.3) | 68 (68.7) |  |
| ***Smoking past/current*** |  |  |  | n/a |
| No | 146 (98.0) | 75 (100.0) | 99 (100.0) |  |
| Yes | 3 (2.0) | 0 | 0 |  |
| ***Alcohol past/current*** |  |  |  | 0.744 |
| No | 116 (77.9) | 58 (77.3) | 80 (80.8) |  |
| Yes | 33 (22.1) | 17 (22.7) | 19 (19.2) |  |

*Data presented as frequency and percentage, and comparison was done using the Chi-square test, otherwise stated. Significant p-values are flagged in bold. ‘a’ means controls differ from DM alone significantly; ‘b’ means controls differ from DM with HTN significantly; ‘c’ means that DM alone differ significantly from DM with HTN ‘p’ means all three groups differ significantly.*

In the case-specific metadata, the median diabetes duration of diabetes for the type 2 DM and HTN group [5, (3-9) years] was higher than the DM alone group [3, (1-7.5) years] ; p=0.011 (Figure 1A). The proportion of DM alone group (72%) on oral antidiabetic medication was higher compared to DM and HTN group (66%); p=0.033 (Figure 1B).


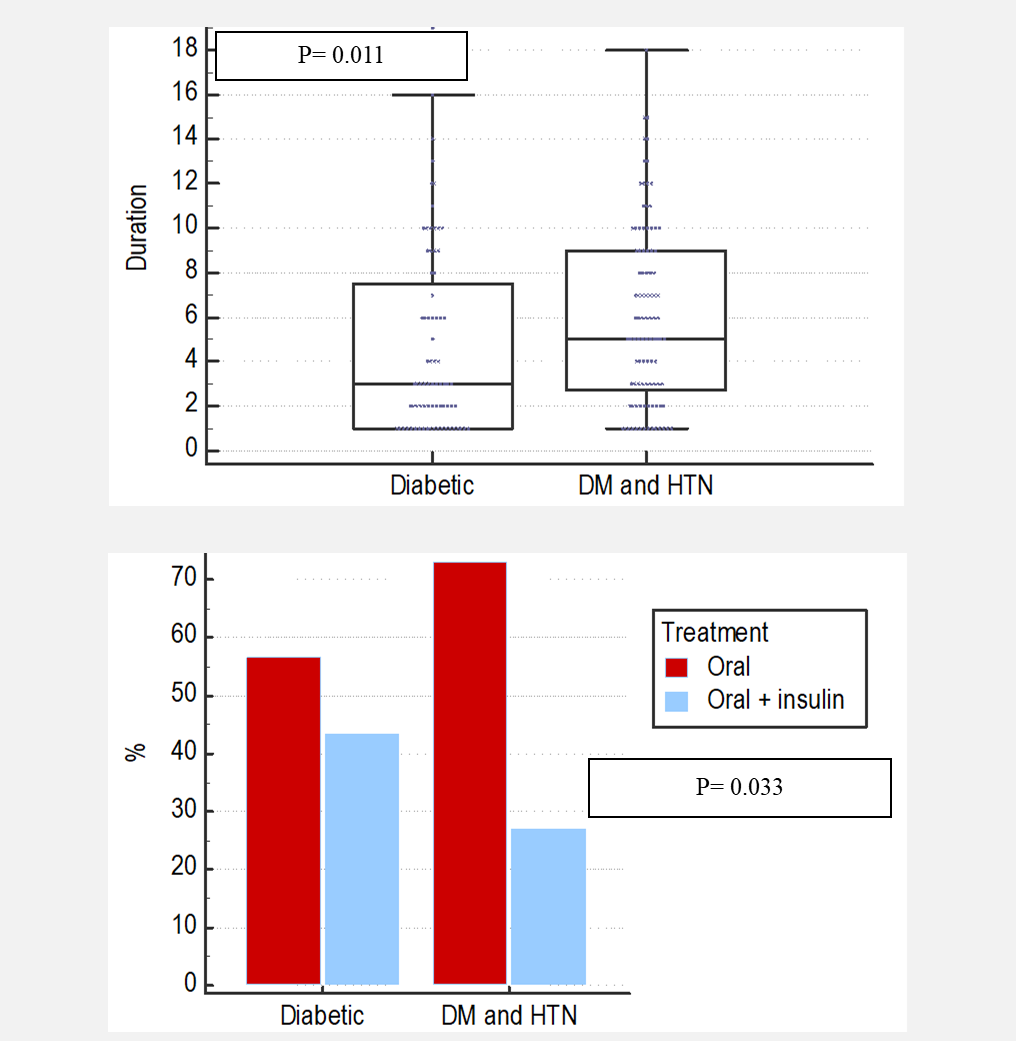


(year)

**B**

**A**

***sF1 A&B: Comparison of treatment duration (A) and treatment option (B) between type 2 DM and 2 DM with HTN groups***

*Data were compared using Mann Whitney test for treatment duration (presented as median, and interquartile range with minimum and maximum values) and Chi-square test for treatment option. Oral denotes oral antidiabetic medications; oral+insulin denotes oral antidiabetic and insulin injection combination.*

**B**
